# Chronset: An automated tool for detecting speech onset

**DOI:** 10.3758/s13428-016-0830-1

**Published:** 2016-12-06

**Authors:** Frédéric Roux, Blair C. Armstrong, Manuel Carreiras

**Affiliations:** 1 School of Psychology, University of Birmingham, College of Life and Environmental Sciences, Birmingham, United Kingdom; 2 Basque Center on Brain, Cognition & Language, San Sebastian, Spain; 3 Department of Psychology, Centre for French & Linguistics at Scarborough, University of Toronto, Toronto, Ontario Canada; 4 Ikerbasque Basque Foundation for Science, Bilbao, Spain; 5 UPV/EHU Universidad del Pais Vasco, Leioa, Spain

**Keywords:** Speech onset, Reading aloud, Automatic detection, Spectral analysis, Optimization

## Abstract

The analysis of speech onset times has a longstanding tradition in experimental psychology as a measure of how a stimulus influences a spoken response. Yet the lack of accurate automatic methods to measure such effects forces researchers to rely on time-intensive manual or semiautomatic techniques. Here we present Chronset, a fully automated tool that estimates speech onset on the basis of multiple acoustic features extracted via multitaper spectral analysis. Using statistical optimization techniques, we show that the present approach generalizes across different languages and speaker populations, and that it extracts speech onset latencies that agree closely with those from human observations. Finally, we show how the present approach can be integrated with previous work (Jansen & Watter *Behavior Research Methods*, 40:744–751, 2008) to further improve the precision of onset detection. Chronset is publicly available online at www.bcbl.eu/databases/chronset.

**Electronic supplementary material:**

The online version of this article (doi:10.3758/s13428-016-0830-1) contains supplementary material, which is available to authorized users.

Reaction time (RT) experiments have a longstanding history in experimental psychology and have been instrumental to achieving several fundamental insights into human cognition (Donders, 1868; Posner & Mitchell, 1967; Sternberg, 1966; Stroop, 1935). Although RTs are classically measured as the time needed to execute a button press, another useful technique consists in measuring the time required to produce spoken responses. Given the ubiquity of speech in human behavior, this approach offers a natural way of measuring response latencies.

To assess speech onset, the current gold standard is to rely on human raters, often aided by semi-automatic rating techniques (Jansen & Watter, 2008; Protopapas, 2007). To date, this approach has yielded some of the most accurate and consistent estimates of speech onsets. Nevertheless, this approach is suboptimal in several respects. First, it is extremely time-consuming, because human raters have to process the waveforms of vocal recordings on a trial-by-trial basis. Second, although agreement levels among raters are typically relatively high, they are prone to subjective bias and other sources of measurement error (Green & Swets, 1966; Morrow, Mood, Disch, & Kang, 2016). Consequently, a considerable amount of work has been dedicated to developing fully automated approaches (Bansal, Griffin, & Spieler, 2001; Jansen & Watter, 2008; Kawamoto & Kello, 1999; Protopapas, 2007).

Hardware-based methods for onset detection, such as voice-key devices, operate by detecting the point in time at which sound pressure levels exceed a given threshold (Rastle & Davis, 2002). In the absence of noise or nonspeech sounds (e.g., lip smacking, respiration, or coughing), voice keys can produce accurate measurements if the experiments are carefully controlled (Korvorst, Roelofs, & Levelt, 2006; Meyer & van der Meulen, 2000; Roelofs, 2005). However, in the majority of cases voice keys fail to achieve accurate measurements whenever vocal responses are preceded by loud noise sounds or are characterized by complex acoustic onsets (Kessler, Treiman, & Mullennix, 2002; Rastle & Davis, 2002), both of which are frequent occurrences in natural speech.

With the ubiquity of personal computers, several software-based solutions have been developed to improve semi- and fully automatic speech onset detection, thereby providing a novel framework for the automatic assessment of speech onset times (Bansal et al., 2001; Donkin, Brown, & Heathcote, 2009; Jansen & Watter, 2008; Kawamoto & Kello, 1999). One important limitation of these approaches, however, is that speech onset is estimated on the basis of a sustained elevation of sound amplitude over time, which in fact can also be triggered by loud nonspeech sounds (Rastle & Davis, 2002). This causes amplitude-based approaches to produce a large number of spurious speech onset estimates, thereby decreasing the overall reliability of these techniques. To overcome this limitation, recent work has focused on estimating speech onset from multiple acoustic features, to make automatic algorithms more robust against noise (Jansen & Watter, 2008; Kello & Kawamoto, 1998). However, despite the significant improvements that have been achieved by adding multiple features to speech onset detection, the accuracy achieved by current hardware and software has remained below the precision of human raters.

Here we present Chronset, a fully automated technique aimed at further enhancing the robustness and accuracy of automatic speech onset detection. The present approach is inspired by previous work in songbirds showing that vocalized sounds have a rich harmonic structure that is absent in noise or nonvocal sounds (Tchernichovski, Nottebohm, Ho, Pesaran, & Mitra, 2000). Building on these findings, we hypothesized that human speech onset and noise sounds have distinct spectral signatures on the basis of which they can be distinguished from each other. We extracted multiple spectral features from audio waveforms, and signaled a speech onset if four different features were simultaneously above a set of threshold levels. To estimate a set of speaker and language-independent threshold parameters, we used an optimization procedure to tune the thresholds for a broad range of waveforms sampled from two laboratories where spoken responses were recorded in two different languages and experimental contexts (Jansen & Watter, 2008; Sadat, Martin, Alario, & Costa, 2012).

Our findings show that Chronset detects voice onset with a high degree of precision relative to human ratings, such that most of its errors occur in a small (<50 ms) window surrounding a manually annotated RT. On the basis of Monte Carlo simulations, we show that the size of Chronset’s estimation error does not substantially reduce the statistical power of experimental data analyses for experiments with standard sample sizes. Furthermore, we show how our approach can be combined with the SayWhen algorithm to achieve estimates of speech onset that are even closer to human rater precision. The present work therefore demonstrates a novel approach for the automatic extraction of speech onset times that provides a substantial improvement over previously reported techniques.

## Method

### Human onset detection

To assess the algorithm’s performance, two previously published datasets were analyzed in the present study: one dataset comprised waveforms in the Spanish language (hereafter, the *Spanish dataset*; Sadat et al., 2012) and a second dataset comprised waveforms in the English language (hereafter, the *English dataset*; Jansen & Watter, 2008). For each dataset, speech onset latencies were calculated by a group of human raters. For the Spanish dataset analyzed in the present study, the data from two raters who used the CheckVocal software (Protopapas, 2007) were already available. In the case of the English dataset, ratings from one individual were supplemented by the data from two additional raters who manually identified onsets after splitting the continuous recording of the audio from the entire experiment for each participant into separate waveforms for each trial. The two additional raters used the Audacity software to measure onsets using a combination of listening, waveform inspection, and spectrogram inspection, whereas the original rater used the SayWhen software (Jansen & Watter, 2008).

### Spectral analysis and acoustic features

**Fig. 1:**
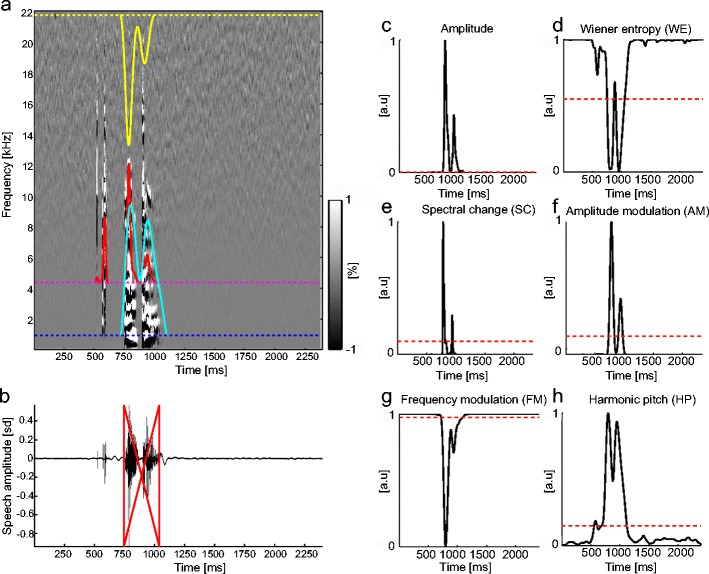
The sound features. (a) Spectrogram of the frequency derivatives of spectral power, where *x*-axis is time and the *y*-axis is frequency. The pseudocolor code represents the sign of the spectral derivatives along the time-versus-frequency plane (black = reduction in power along the *y*-axis, white = increase of power along the *y*-axis). The individual thresholds (dotted lines) and thresholded time series (solid lines) of three acoustic voice features are shown on top of the spectrogram (yellow = frequency modulation [FM]; red & magenta = amplitude; blue & cyan = harmonic pitch [HP]). (b) Raw voice signal in the time domain. The red solid lines mark the on- and offsets of voice sounds measured by the algorithm. Note that the large-amplitude prevocal sound that occurs close to the actual onset of speech is not classified as speech because several features, including FM and HP, remain below threshold levels. *x*-axis = time, *y*-axis = standardized amplitude (*z* score). (c–h) Acoustic features of speech obtained from the various spectral estimates (black solid lines) and the individual feature thresholds (red dotted lines). *x*-axes = time in milliseconds, *y*-axes = normalized feature magnitude

Speech onset detection in Chronset relies on six basic acoustic features of speech sounds. To derive these six features, we first computed a time–frequency spectrogram as well as its derivatives over time and frequency, based on the raw waveform (Figs. 1a–h). Spectrograms were computed using multitaper spectral analysis, which is similar to standard spectral analysis but produces more robust parameter estimates; it also allowed us to compute the derivatives of the spectrogram and to extract the features of harmonic activity (Percival & Walden, 1993; Tchernichovski et al., 2000; Thomson, 1982). The multitaper analysis in the present study involved a sequence of *k* = 8 discrete prolate spheroidal tapers, a window length of 10 ms, and a step size of 1 ms. As a result, the spectral concentration of energy for each frequency bin had a half-bandwidth of ±0.5 kHz. The choices of the parameters for window length and step size values were based on previous work in which similar parameter values were employed to study the development of rhythmic structure in bird song (Saar & Mitra, 2008).

Unless stated otherwise, all features were normalized to range from 0 to 1 for each sound recording, with 0 and 1 corresponding to the minimum and maximum values of each feature. Gaussian smoothing over time (10 ms) was applied to all voice features, thus ensuring that only large, systematic changes in the feature would be used in determining the voice onset. A brief description of each feature follows below (for additional details, see Tchernichovski et al., 2000). These and all subsequent analyses were carried out using the Chronux toolbox (http://chronux.org/; Bokil, Andrews, Kulkarni, Mehta, & Mitra, 2010), as well as custom scripts written in MATLAB (The Mathworks).

#### Amplitude

The amplitudes of the speech sounds were computed as the logarithm of spectral power integrated over frequencies between 0.15 and 22.05 kHz (Fig. 1c). Speech sounds typically have higher amplitude than noise, and thus provide an indicator of speech onset. However, other sounds (e.g., coughing, lip smacking) have high amplitude as well, thereby reducing the reliability of this feature for speech onset detection.

#### Wiener entropy (WE)

Entropy is a measure of how random versus organized a signal is. In the context of voice sounds, it can be applied to measure whether a sound is occurring relatively uniformly across the entire spectrum, or only in specific frequency bands. Noise sounds tend to have a flat spectrum, whereas the spectrum of voiced sounds will be organized into clear peaks. Many different mathematical formulations of entropy have been developed, which all relate to these same principles (Shannon, 1948). Here, we used WE, which is computed as the ratio of the geometric mean and the arithmetic mean of the spectrum (Tchernichovski et al., 2000). Importantly for the present purposes, WE is amplitude-independent, and thus is not affected by the distance of the speaker from the recording device, the speaker’s overall loudness, or the absolute signal-to-noise ratio (Fig. 1d).

#### Spectral change (SC)

SC is a combined measure of how power changes simultaneously in both time and frequency (Fig. 1e). Noise typically shows less spectral change than do voiced sounds.

#### Amplitude modulation (AM)

AM is the overall change in power across all frequencies, and thus reflects the magnitude of the change in energy of a speech sound over time (Fig. 1f). Voiced sounds will tend to modulate amplitude more strongly than other sounds, such as lip smacks, which typically generate a very transient sound.

#### Frequency modulation (FM)

FM assesses how much the concentration of power changes across spectral bands over time (Fig. 1g). The energy of tonal sounds tends to remain concentrated within specific spectral bands (low FM), whereas noise typically has a rapidly changing, nonconstant concentration of power across frequencies (high FM).

#### Harmonic pitch (HP)

HP estimates the spectral structure of harmonic sounds. To estimate HP, we computed a second spectrum of the power spectrum, which measures the periodicity of the peaks in the power spectrum (Bogert & Healy, 1963; Oppenheim & Schafer, 1989) for each time point (Fig. 1h). A high value of HP signifies that the acoustic signal is composed of harmonically related frequencies. In contrast, low HP indicates the absence of harmonic structure. Voiced sounds, which typically contain a collection of harmonics (formants), will show a high level of periodicity in the power spectrum (Noll, 1967), whereas the spectrum of noise is typically characterized by the absence of resonance.

### Automatic detection criteria

To improve the robustness of our algorithm against loud noise sounds, speech onset was detected if four of the six features were simultaneously elevated above threshold levels for 35 ms. Furthermore, to ensure that our algorithm could detect unvoiced onsets such as [s], [f], or [p], we quantified speech onset as the first point in time at which the amplitude was elevated above threshold within the time window defined by the four-feature criterion. Because the algorithm uses a low threshold for the amplitude feature, this additional criterion allowed us to detect low-amplitude unvoiced sounds that often precede voiced sounds but do not have a harmonic spectrum.

### Statistical tuning of optimized feature thresholds

To identify a set of thresholds that provided good sensitivity and validity across waveforms, we estimated the thresholds for each individual feature using a customized version of the gradient descent algorithm (Hinton & Sejnowski, 1986; see also Armstrong, Watson, & Plaut, 2012). Feature thresholds were optimized on the basis of vocal responses recorded in the Spanish dataset, which comprised 150 waveforms per participant (*n* = 14) for which the individual voice onsets were estimated by two human raters using semi-automatic techniques (total number of analyzed waveforms: 2,100). To ensure that the optimized feature thresholds were not overfitted, we randomly divided the waveforms of the Spanish dataset into a training set (80 % of the data) and a testing set (20 %). Optimization was performed on the training set, whereas the testing set was used to assess the accuracy achieved by the optimized feature thresholds. In both sets, the fit associated with a particular set of feature thresholds was quantified by measuring the maximum likelihood estimate of the standard deviation (*SD*) of the regression residuals, which assessed the difference between the individual hand-coded onset latencies and the fitted regression line. A poor fit of the estimated thresholds was thus associated with a higher *SD* for the regression residuals, whereas a better fit was associated with a lower *SD*. By selecting those thresholds that lowered the overall *SD* of the test data, it was possible to optimize the thresholds such that the returned automatic estimates of speech onset were in close agreement with those of the human ratings across a broad range of waveforms (for more details, see the supplemental information). The optimization algorithm stopped either after 1,000 attempts to modify the thresholds or if 50 consecutive attempts failed to improve the fit. To maximize the likelihood that the best possible thresholds would be identified, we repeated this optimization process for 100 randomly chosen partitions of the waveforms into training and test sets. The final thresholds that we selected were those associated with the smallest *SD* and the largest *R* ^2^ on the testing data.

### Dependent measures of algorithm performance

To assess the performance of both Chronset and the other speech onset detection algorithms, we evaluated each algorithm using two dependent measures: (1) absolute-difference scores and (2) regression fits in terms of *R* ^2^ and regression residuals.

#### Absolute difference (AD):

The AD reflects the absolute difference between the automatic and manually estimated speech onset latencies and is computed as

$$ A{D}_{(i)}={y}_{(i)}{\textstyle \hbox{-} }{x}_{(i)}, $$

where *y*_(*i*)_ is the manual rating of speech onset for a single waveform *I*, and *x*_(*i*)_ is the corresponding automatic estimate. ADs are currently used as the gold standard to quantify the precision of automatic speech detection, and we report both a “tight” measure of fit (automatic estimates within 10 ms of the manual estimates) and the characteristics of the cumulative distribution of ADs (for a similar approach, see Jansen & Watter, 2008; Lin & Wang, 2011; Sonderegger & Keshet, 2012)*.*

#### *Regression fits (r*^2^*and regression residuals)*:

The regression fit quantified how well the regression line fit the unobserved linear relationship between the automatic estimates and manual ratings. Note that *R* ^2^ is more sensitive to large deviations from the optimal fit than are ADs, and is less sensitive to small deviations from the regression line, because differences are squared. These properties are a strength of the regression measure, because they highlight whether an algorithm generates large numbers of outliers, and because they eliminate any measurement bias that can be attributed to either a rater’s perceptual bias (Green & Swets, 1966; Morrow et al., 2016) or an algorithm´s systematic measurement error (for more details, see Figs. S1–S4 in the supplement materials).

Indeed, linear regression offers a way to quantify measurement bias, by estimating the linear relationship between the automatic scores *X*_(*i*)_ and the manual scores *Y*_(*i*)_ for each audio waveform *i* as

$$ {Y}_{(i)} = \beta 0 + \beta 1{X}_{(i)}+{e}_{(i)}, $$

where *β*0 represents the intercept of the regression slope *β*1, and *e*_(*i*)_ represents the measurement error. Because *e* is the population error, which is not directly observable, the sample’s measurement error is computed as

$$ {\widehat{e}}_{(i)}={y}_{(i)}^{\hbox{'}}{\textstyle \hbox{-} }{Y}_{(i)}, $$

where *ê*_(*i*)_ is the sample’s measurement error or regression residual, *y'*_(*i*)_ is the predicted manual score, and *Y*_(*i*)_ is the actual observed manual score. Thus, the regression residuals reflect the amounts of unobserved error that can be attributed to either the manual or the automatic scores.

One important limitation of using regression statistics to evaluate algorithm performance, however, is that a good fit (high *R* ^2^) will not necessarily mean that the absolute values of the onsets are identical. Rather, it means that relative changes in the manual onsets are reflected by highly systematic changes in the automatic onsets. Thus, here we argue that an optimal algorithm should maximize performance on a new composite measure that minimizes ADs and maximizes regression fit.

### Algorithm benchmarking

To benchmark the accuracy of Chronset and the other previously reported algorithms, we first compared the performance of Chronset against two frequently employed techniques: Epd (Bansal et al., 2001) and CheckVocal (Protopapas, 2007). In the case of CheckVocal, which was originally designed for semi-automatic onset detection, the onset latencies were accepted without visual inspection, so the reported performance of CheckVocal does not reflect the accuracy of semi-automatic analyses.

### Cross-validation of optimized feature thresholds

We cross-validated the thresholds optimized on the basis of the first dataset (Spanish dataset) by testing the robustness of these same thresholds on a second set of audio recordings obtained from a sample of waveforms recorded in English (English dataset). These new waveforms had been used previously to develop and assess the reliability of the SayWhen onset detection software (Jansen & Watter, 2008). In total, this dataset comprised approximately 167 trials per participant (*n* = 22), from which voice onsets were identified manually by three human raters as well as by the SayWhen algorithm (total number of analyzed waveforms: 3,674). This second dataset thus provided a benchmark against which we examined the robustness of the optimized thresholds for influences specific to different languages and speakers, as well as to recording equipment-related influences. It also enabled us to compare our results to those from the SayWhen algorithm.

### Human onset detection and interrater variability

Systematic differences between the individual raters were measured by using the intraclass correlation (ICC). Given that all raters independently coded all trials, the ICC was computed using a two-way mixed-effects model of the single-trial voice onset latencies (Shrout & Fleiss, 1979). According to this approach, an ICC value of 0 reflects the absence of agreement—that is, all scores differed systematically across raters—whereas an ICC value of 1 reflects perfect agreement between the raters. The different datasets employed a combination of ratings from new raters and the original ratings from the published research. In the case of the English dataset, raters segmented the raw continuous audio file into individual waveforms for each trial (for more details on the original data, see Jansen & Watter, 2008).

### Detection performance for distinct phonetic onset categories

To examine how the onset detection performance of Chronset may be affected by distinct phonetic onset types, such as unvoiced consonants, which are more challenging to detect than voiced vowels, we carried out a separate analysis in which we split the data from the English dataset into different groups of waveforms that corresponded to different phonetic onsets. The phonetic code of each individual waveform was determined by two human raters who listened to each waveform and then selected the corresponding phonetic onset code from the Carnegie Mellon University Pronouncing dictionary (www.speech.cs.cmu.edu/cgi-bin/cmudict#about). We then examined the fit in terms of the AD and regression fits for each phonetic onset for both Chronset and SayWhen.

## Results

### Agreement between human ratings for the speech recordings in the Spanish dataset

**Fig. 2:**
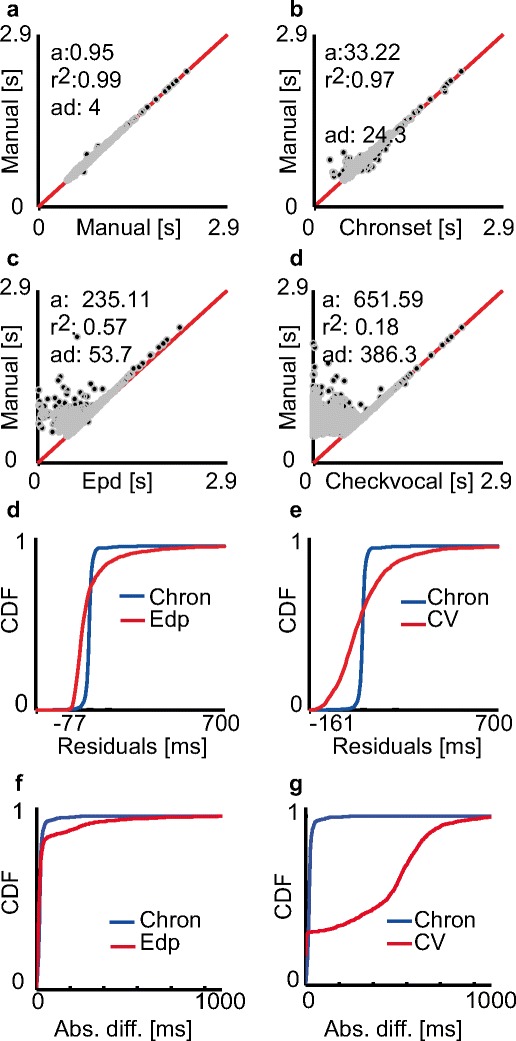
Comparisons of pooled manual and automatic measurements in the Spanish dataset. (a) Regression fit between the human ratings. The dark dots correspond to the individual onset latencies, and the red solid line reflects the line of identical correspondence. *x*-axis = latencies hand-coded by Rater 1; *y*-axis = latencies hand-coded by Rater 2. (b–d) Regression fits between different automatic estimates and the manual ratings. The same conventions are applied as in panel a. *x*-axes = automatic latencies, *y*-axes = average of hand-coded latencies. (e & f) Solid lines represent the cumulative density functions (CDFs) of regression residuals for the different automatic methods. Chronset is always depicted in blue, and the alternative algorithms in red. (g & h) Analogous plots to panels e and f, but for AD scores. Note that the *x*-axes in panels e–h have been scaled differentially to emphasize the differences between the algorithms

We first examined the correspondence between the hand-coded onset latencies calculated using a semi-automatic method by the two human raters for vocal responses in the Spanish dataset (Fig. 2a). This revealed that the hand-coded onset latencies were highly consistent and strongly correlated between the human raters (ICC = .99, *R* ^2^ = .99, offset = 0.95 ms). Accordingly, large proportions of the AD scores (100 %; *SD* = 0.26 ms) and regression residuals (98.2 %; *SD* = 8 ms) remained within the ±10 ms range. This very high level of agreement was likely due to the semi-automatic method employed by both raters, who could accept a fit suggested by CheckVocal and thus produce exactly the same onset estimates.

### Comparison of automatic speech onset detection for the speech recordings in the Spanish dataset

**Fig. 3:**
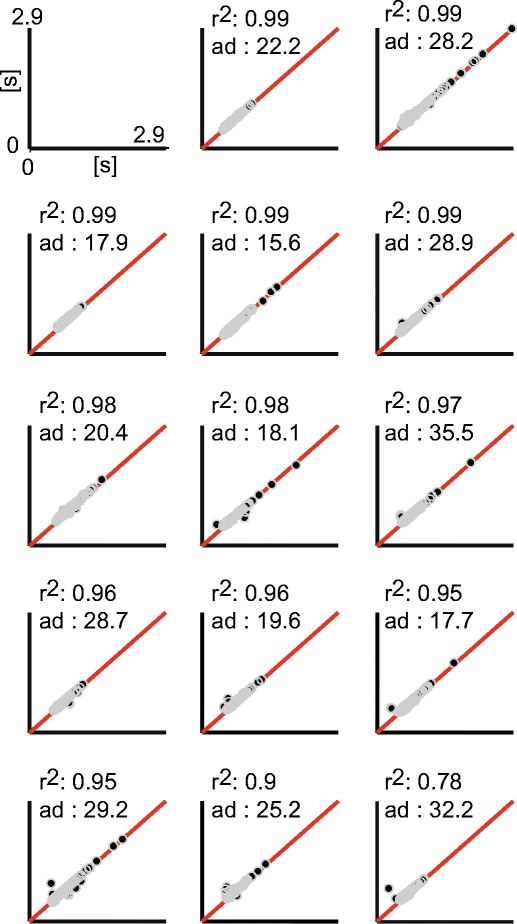
Comparison of manual and automatic measurements for individual speakers in the Spanish dataset. The black dots represent the individual measurements, and the red solid lines represent the lines of identical correspondence. Same conventions are applied as in Figs. 2b–d

We observed a strong linear relationship between the human ratings and the automatic scores as measured by Chronset (*R* ^2^ = .97, offset = 28 ms; Fig. 2b). This strong linear relationship was observed across the entire range of participants (mean *R* ^2^ = .95, *SD R* ^2^ = .05; Fig. 3). Furthermore, the proportions of regression residuals and AD scores within the ±10 ms range corresponded to 63 % (*SD* = 30 ms) and 28 % (*SD* = 56 ms), respectively. As is depicted in the cumulative density functions, the majority of the automatic estimates achieved by Chronset occurred in the 10 to 50 ms range, relative to the human ratings, with virtually no outlier misestimations outside of this range (Figs. 2e–h).

Lower correspondences between the automatic and manual scores were observed for the two other examined algorithms. The proportions of regression residuals that were within the ±10 ms range remained below 10 % for both Epd and CheckVocal (Epd, 8 %, *SD* = 118.8 ms; CheckVocal, 6 %, *SD* = 164 ms; Figs. 2e and f), which was considerably lower than Chronset’s performance. The tendency for these algorithms to misestimate the regression residuals persisted across the entire distribution, as is shown in these figures and reflected in the poor summaries of the fits via *R* ^2^ (Epd, *R* ^2^ = .57, offset = 235 ms; CheckVocal, *R* ^2^ = .18, offset = 651 ms; Figs. 2c and d). The proportions of AD scores that remained below 10 ms were fractionally higher than with Chronset for both CheckVocal (30 %; Fig. 2g) and Epd (33 %; Fig. 2h) than for Chronset. However, assessment of the cumulative density functions revealed that these algorithms produced highly variable misestimations throughout the 0 to 1,000 ms range. This is reflected by the standard deviation of the difference scores, which showed four to ten times more overall variability for these algorithms than for Chronset (*SD*s: CheckVocal = 313 ms, Epd = 129 ms). These algorithms were thus fractionally better at estimating latencies within the 10 ms window, but when they failed to do so, the misestimations were substantial. These deviations appear to be attributable primarily to false early detections.

### Agreement between the human ratings for the speech recordings in the English dataset

**Fig. 4:**
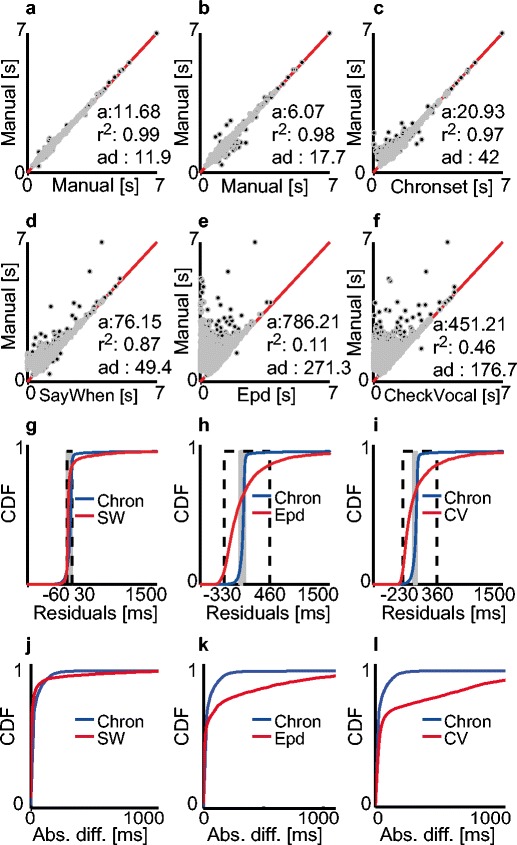
Comparisons of pooled manual and automatic measurements in the English dataset, following the same conventions as in Fig. 2. (a, b) Regression fits between the human ratings. The dark dots correspond to the individual onset latencies, and the red solid lines are the regression lines. (a) Relationship between Raters 1 and 2. (b) Relationship between the average ratings of Raters 1 and 2 and the manual ratings of Rater 3. (c–f) Regression fits between various automatic estimates and the manual ratings. (g–i) Cumulative density functions (CDFs) of regression residuals for the different automatic methods. (j–l) CDFs of AD scores for the different automatic methods. Scaling of the *x*-axes in panels e–h follows the same conventions as in Fig. 2

The robustness and validity of the optimized feature thresholds was assessed by computing the linear regression between the human ratings and the onset latencies estimated by Chronset in the English dataset. The onset latencies estimated by three human raters in the English dataset were highly consistent across raters, and therefore provided a reliable benchmark against which to assess the robustness of the optimized thresholds (ICC = .99, *R* ^2^ = .98, offset = 9 ms; Figs. 4a and b). The regression residuals that remained within the range of ±10 ms corresponded to 72 % of the data (*SD* = 44 ms), whereas only 70 % of the AD scores remained below 10 ms across the three raters (*SD* = 2 ms). In practical terms, the variability in the manual onset measurements (vs. the semi- automated onsets generated by the human raters in Spanish) provides a better quantification of the variability between raters when applying the gold standard manually. In turn, this shows why the variability in human raters is becoming an increasingly important issue, and why a gold standard based on the ADs from human ratings is lacking in some respects.

### Comparison of automatic accuracy levels for the speech recordings in the English dataset

**Fig. 5:**
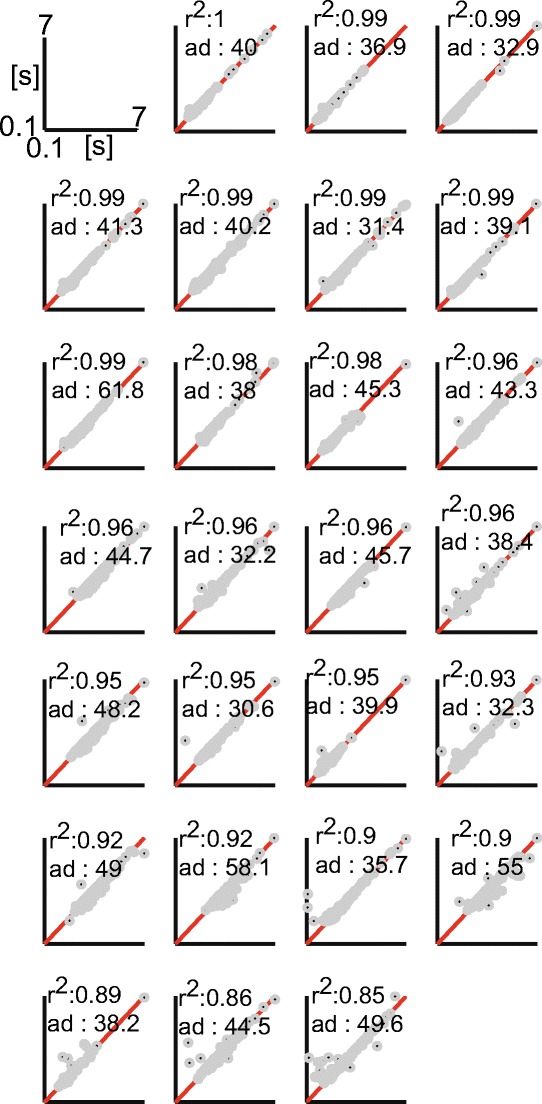
Comparison of manual and the automatic measurements for individual speakers in the English dataset. The same conventions are used as in Fig. 3

We observed a strong linear relationship between the average human ratings and the automatic latencies estimated by Chronset over the pooled waveforms in the English dataset (*R* ^2^ = .97, offset = 21 ms; proportion of regression residuals within ±10 ms range = 26 %, *SD* = 90 ms; Figs. 4c, g and i). The AD scores showed that Chronset produced 24 % of its automatic responses within 10 ms of the manual responses (Figs. 4c, j, and l). Inspection of the cumulative density functions indicated that the bulk of Chronset’s misestimations occurred within 50 ms of the human RT, with very few outliers. The high quality of the fits was also observed at the level of individual participants (mean *R* ^2^ = .96, *SD R* ^2^ = .03; Fig. 5). Collectively, these results show that the thresholds derived from the optimization in the Spanish dataset enabled Chronset to perform well in the English dataset.

Turning to the other algorithms, the correlations were substantially lower between the manual ratings and the automatic scores estimated by all of the other algorithms examined (SayWhen, *R* ^2^ = .87, offset = 76 ms; Epd, *R* ^2^ = .11, offset = 786 ms; CheckVocal, *R* ^2^ = .45, offset = 451 ms; Figs. 4d–f). In addition, when compared to Chronset, lower proportions of the regression residuals were observed within the ±10 ms range for all of the other algorithms (SayWhen: 6 %, *SD* = 172 ms; Epd: 2 %, *SD* = 454 ms; CheckVocal: 3 %, *SD* = 356 ms; Figs. 4g–i). The higher accuracy of Chronset persisted when we examined the full distributions of regression residuals, and not only those falling within the 10 ms window. Turning to the AD scores, a different pattern of results emerged: Both SayWhen and Epd produced more AD scores within 10 ms of the human rating (64 % for SayWhen, 54 % for Epd), whereas CheckVocal produced only 17 % of its RTs in this window (Figs. 4j–l). This indicates that both SayWhen and Epd are capable of producing very precise RTs on a substantial number of trials. However, inspection of the full cumulative density functions showed that all three algorithms also produced more outlier misestimations outside the 50 ms range than did Chronset, particularly in the cases of Epd and CheckVocal. Thus, the extreme sensitivity to real speech onset displayed by these other algorithms is accompanied by an increased likelihood to be triggered prematurely by nonspeech sounds.

### Comparison of speech onset detections for distinct phonetic onset categories

**Fig. 6:**
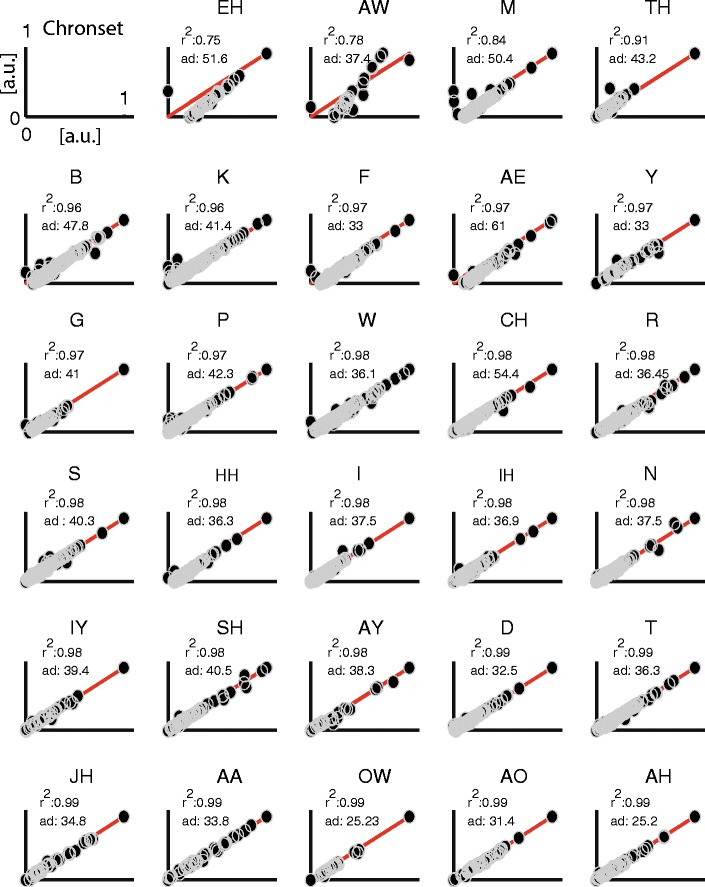
Speech detection performance of Chronset for different phonetic onsets. Each individual panel represents the fit achieved by Chronset for a different phone. The phone set is based on the adapted ARPAbet used in the Carnegie Mellon Pronouncing Dictionary (www.speech.cs.cmu.edu/cgi-bin/cmudict)

**Fig. 7:**
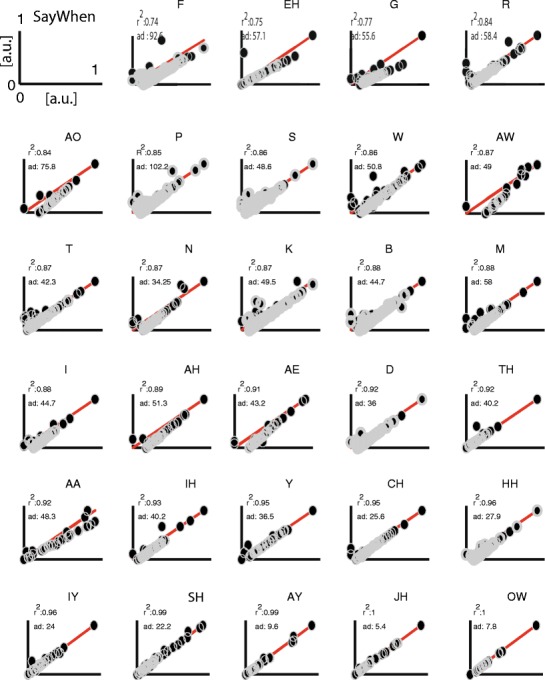
Speech detection performance of SayWhen for different phonetic onsets. The same conventions are used as in Fig. 6

To examine whether the performance of Chronset varied as a function of different phonemes, and hence whether some of the residual error in our regression fits could be attributable to issues with particular onsets, we subdivided our data on the basis of the onset phoneme. Figure 6 plots these data for Chronset, and Fig. 7 plots the analogous data for SayWhen. Each individual plot also reports the *SD* of the AD scores and the *R* ^2^ measure of fit.

On the basis of inspection of the Chronset data for regression residuals, it is clear that estimating a few of the onsets (EH, AW, M, and TH) was more difficult, notwithstanding that performance was nevertheless still relatively good (*R* ^2^ range for these onsets: .75–.91). Performance for all of the other onsets was near ceiling (all *R* ^2^s ≥ .96). Similarly, AD scores tended to decrease as *R* ^2^ increased, although there were many small and a few more substantial rearrangements in performance. Of particular note are that, again, four phonemes showed notably worse performance than the others, with *SD*s of the AD scores >50 ms. Two of these items were also associated with the poorest *R* ^2^ scores (EH and M), but the two others were not (AE and CH), although they still fell in the lower half of all observed *R* ^2^ scores.

As compared to Chronset, SayWhen had a similar overall range of performance across all phonemes in terms of the regression fits (*R* ^2^ range: .74–1.0). However, there was more variability and gradation in how well SayWhen was able to predict onsets for the different phonemes: Whereas Chronset only had three phonemes with *R* ^2^ fits below .9, SayWhen had 16 such phonemes. Similarly, whereas Chronset had only four phonemes for which the *SD* in the overall AD scores was >50 ms, SayWhen had eight such phonemes.

These results suggest that the better performance for Chonset in overall *R* ^2^ and its different distribution of AD scores is largely attributable to better fits for many of the phonemes that were most challenging for SayWhen (the fact that SayWhen still performed well on those phonemes notwithstanding). These results also point to two practical implications: On the one hand, they provide guidance for which types of onsets should be targeted in future improvements to automatic onset detection algorithms. On the other hand, these results suggest that insofar as some experimental conclusions can be derived without relying on the four most problematic onsets for Chronset, there should be an even smaller difference between manual onset detection and our automated approach.

## Discussion

In the present article, we report an approach that permits the automatic detection of speech onsets in audio recordings of human voice recordings, thereby allowing this measure to be used by researchers studying cognitive and perceptual processes including, but not limited to, speech (Coltheart, Rastle, Perry, Langdon, & Ziegler, 2001; Donders, 1868; Plaut, McClelland, Seidenberg, & Patterson, 1996; Posner & Mitchell, 1967; Sternberg, 1966; Stroop, 1935). The present approach represents a novel technique for the automatic detection of speech onsets in audio recordings of human speech. It demonstrates that the onset latencies identified by Chronset are substantially more robust than those produced by other popular algorithms, particularly in terms of avoiding outliers, at the expense of a small degree of fine-grained precision relative to an alternative algorithm, SayWhen. It also shows that our (and other) approaches produce measurements that are rapidly converging on “gold standard” human levels of precision. Related to this point, we also observed nontrivial variability in our raters’ generation of manual onsets—particularly with respect to the absolute value of the onset. Collectively, these observations indicate that a more rigorous consideration of the gold standard is warranted when comparing automated and manual onset detection in the future. This could include efforts to better estimate the gold standard by extracting the latent “true” onset by using dimensionality reduction, as we have done in our analyses. Similarly, it could include updating the gold standard to a composite measure, which would include both ADs and regression fits.

### Advantage of using multiple features in speech onset detection

Threshold-dependent measures that estimate voice onset from fluctuations in the amplitude level of speech waveforms often fail to detect complex onsets of speech (Kessler et al., 2002; Rastle & Davis, 2002). Similarly, these techniques frequently produce false alarms if high-energy nonvocal or prevocal sounds occur prior to actual vocal responses (Rastle & Davis, 2002). Our results show that the combination of multiple features achieves higher accuracy levels than do approaches that detect voice onset only on the basis of amplitude fluctuations (albeit at the expense of a small amount of fine-grained precision). This claim is also supported by the substantially higher proportion of overall variability in human measurements that is explained by Chronset estimates but not by other algorithms, by the reduction in variability across different onset phonemes, and by the smaller set of phonemes whose estimation performance was not near ceiling. These differences in performance are likely due to the fact that amplitude is sensitive to absolute sound levels, and therefore cannot reliably discern loud nonspeech from genuine speech sounds, whereas a combination of features (some of which are amplitude-independent) can.

### Optimization and cross-validation of feature thresholds in a different language and experimental setting

To derive a set of thresholds that could be well-suited for application to a broad range of waveforms and yield accurate onset estimates, we used statistical optimization (Papadimitriou & Steiglitz, 1982) and tuned the thresholds for each individual feature by minimizing the *SD* of the regression residuals. This allowed us to identify a set of thresholds that were robust against speaker specific differences and that achieved accuracy levels near identical to human observations. Moreover, we assessed the basic validity of our method by testing our optimized thresholds on a second novel dataset, which comprised speech waveforms recorded in a different language using different equipment and another task that required spoken responses. Together, these results support the robustness of our optimized thresholds against differences in language and lab equipment.

### Toward a composite gold standard for algorithm benchmarking: Integrating insights from difference scores and regression fits

Differences between manual ratings and automatic scores have been reported in the literature to quantify the measurement error of automatic speech onset detection (Jansen & Watter, 2008). This measure has clear theoretical value, but is also limited in that it may be confounded in some cases by the measurement bias of human raters, as demonstrated in our own comparisons of interrater reliability. This variability in the thresholds at which human raters visually detect the onset of speech in audio waveforms can distort the error attributed to algorithm performance. For instance, if a human rater systematically rates the true speech onset with an offset of 10 ms, the mean deviation of the algorithm from human ratings might be shifted by 10 ms incorrectly, which can work either for or against the algorithm, depending on that algorithm’s own potential biases.

To circumvent this problem, in the present study we utilized regression residuals as a second measure to benchmark the performance of Chronset against other algorithms. Regression residuals measure the difference between automatic scores and the regression line, which represents the unobserved relationship between the automatic scores and the onset of speech. The regression line itself is fitted by minimizing the differences between the regression line and the manual and automatic scores (here using maximum likelihood estimation), thereby automatically minimizing the contribution of systematic bias in each variable (Cohen, Cohen, West, & Aiken, 2003). Thus, any consistent bias that was due to the rater or the algorithm would be minimized by the regression residuals, and only those values that did not lie on the regression line would generate a large nonzero value. Regression residuals can therefore be interpreted as a bias-free estimate of measurement error that is due to either the algorithm or the human rater, which is an important advantage over the classic measure based on AD scores.

Regression estimates, however, have their own limitations, in that they are more sensitive to extreme values than they are to small deviations from the regression line. They also lack the simple transparency offered by difference-score measures. Thus, we view the present research as a novel gold standard for evaluating algorithms of speech onset detection by including both difference scores and regression residuals. These measures clearly offer complementary insights into how each algorithm’s responses align with manual responses, and together they can provide more targeted guidance for refining model development.

### Simulation of the effects of measurement error on statistical power

The present analyses show that automatic onsets are approaching the performance of human raters. One important question, however, is how important are the remaining differences in onset estimation for drawing conclusions from an experiment? To estimate how fluctuations in the accuracy of Chronset could potentially impact the outcome of an experiment, we conducted a follow-up Monte Carlo simulation of the effects of measurement error on statistical power. On the basis of previous empirical work, we simulated a within-participants design with 100 observations per participant in which the onset latencies in two conditions differed by a small, but detectable and meaningful, effect size of either 15 or 30 ms (Grainger, 1990; Kello & Plaut, 2000; Sadat et al., 2012). We also simulated different amounts of within-condition variability (*SD* range: 50–125 ms), as well as differences in the sample size (range: 5–60 participants). To simulate variability in the precision of the automatic measurements, we chose the *SD* of the regression residuals from the cross-validation, which corresponded to 87 ms. For each combination of parameters, we simulated 204 experiments and measured the resulting effect size by comparing the means of both conditions for the simulated manual and automatic latencies using Student’s *t* test for dependent samples (alpha = .05, two-tailed). We then computed an index *d* that quantified how different levels of measurement error in our algorithm could reduce statistical power as compared to manual ratings. This index was computed as

$$ di=\left( nt1\kern.1em {\textstyle \hbox{-}}\kern.1em  nt2\right)/nt1 $$

where nt1 is the number of significant *t* values obtained from simulated manual ratings, nt2 is the number of *t* values that yielded a significant effect size based on simulated automatic scores, and *i* indexes the three levels of measurement error used in the present simulation. The index *d* thus reflects the reduction in statistical power caused by an *SD* of measurement errors corresponding to 87 ms.

**Fig. 8:**
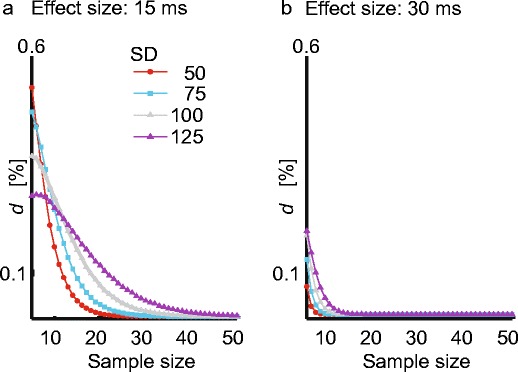
Results of Monte Carlo simulations. Panels a and b represent the effects of measurement error on statistical power as a function of two levels of mean difference between the conditions and multiple levels of within-condition variability

The results of the Monte Carlo simulation showed that the effects of measurement error on the statistical power of an experiment depend on both the magnitude of the measurement error and the sample size (Fig. 8). For simulated measurement errors with an *SD* of 87 ms, the reduction in statistical power remained below 10 % for all samples sizes with *n* > 22, and rapidly approached 0 as the sample sizes increased beyond that point.

### Combining Chronset and SayWhen in a “mixture-of-experts” model

Taken together, the prior set of results highlight that our fully automated voice onset detection algorithm is able to provide sufficiently precise estimates as to have a negligible impact on the analyses of the results of a standard experiment. However, it is clear that in some settings (e.g., experiments involving extremely small numbers of trials or participants), an even more precise estimate of speech onset is important. One clue to how such an improved algorithm can be achieved emerges from a comparison of the *R* ^2^ and raw difference score results. These data highlight that in many ways, Chronset and SayWhen—the next best algorithm in terms of *R* ^2^, and the superior algorithm in terms of AD, particularly in the 10 ms window nearest the human ratings—exhibit complementary patterns of performance. Chronset sacrifices extremely high precision for individual latencies to ensure the robust estimation of relatively precise onsets in the absence of many outlier misestimations—the avoidance of which is especially important for a fully automatic onset detection procedure. In contrast, SayWhen achieves extremely precise estimates at the expense of many outliers, which may be an appropriate compromise in the context of a semi-automatic procedure in which outlier trials can be manually reinspected.

**Fig. 9:**
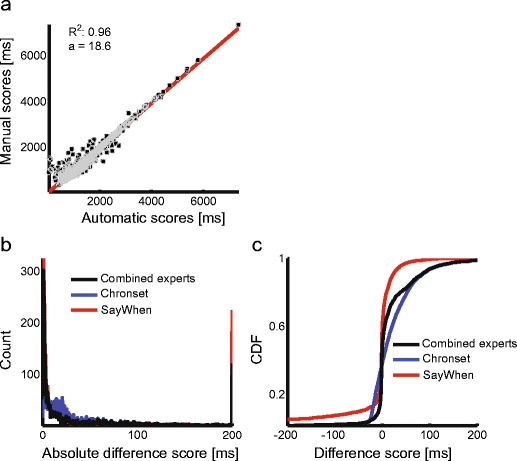
Automatic onset detection based on the mixture-of-experts model. (a) Linear regression fit of manual onset scores for automatic onset latencies estimated on the basis of the mixed-expert model. *x*-axis = automatic scores (in milliseconds); *y*-axis = manual scores (in milliseconds). (b) Histogram of absolute-difference scores. (c) Cumulative distribution function (CDF) of raw difference scores. Color legend: black solid line = mixed experts, blue solid line = Chronset, red solid line = SayWhen

It is possible, however, that an even better fully automatic solution could be achieved by combining these two approaches. This would allow SayWhen to generate extremely precise estimates with a certain probability of a large misestimation, and then have Chronset “reestimate” any potential outliers (for related methods, see Armstrong et al., 2015). To develop such a model, we examined the point on the cumulative distribution of AD scores at which Chronset provides more precise estimates of onset latency than does SayWhen. This was found to occur at approximately 55 ms. We then developed a mixture-of-experts algorithm in which we took the difference between the SayWhen and Chronset latencies, using the SayWhen latencies when the difference between the two algorithms was < 55 ms and the Chronset latencies when the difference was ≥ 55 ms. The results are plotted in Fig. 9, and show that the resulting mixture-of-experts algorithm capitalizes on the strengths of each individual algorithm while avoiding each algorithm’s weaknesses, thereby achieving better estimates than either model alone. This also helps highlight the particular areas of improvement necessary for each individual algorithm, and provides a practical means of achieving unprecedentedly precise fully automatic onset detection in cases in which such precision is essential.

### Related approaches in automatic voice onset time detection and voice activity detection

Voice onset time (VOT) reflects the delay between the beginning of a speech sound and the onset of vocal cord vibration and has been applied to study phonetic perception (Clayards, Tanenhaus, Aslin, & Jacobs, 2008), whereas voice activity detection (VAD) is a technique used in speech processing in which the presence or absence of human speech is detected (Ramírez, Górriz, & Segura, 2007) and has been applied in telecommunication. Recent work in automatic VOT detection has achieved accuracy levels that are similar to human precision levels by combining multidimensional feature extraction from speech signals and machine learning (Lin & Wang, 2011; Sonderegger & Keshet, 2012). Similarly, feature extraction and machine learning techniques have been applied to improve the performance of VAD in several fields (Kim, Chin, & Chang, 2013; Park et al. 2014). These studies therefore support the present approach toward the automatic extraction of onset latencies from human speech in the context of behavioral experiments. Moreover, the Chronset algorithm may help inform algorithms designed for automatic VOT detection, given that VOT measurements depend on the accurate detection of speech onset latencies (Das & Hansen, 2004).

### Conclusion and outlook for Chronset

Our data show that multiple features can enhance the accuracy of automatic speech onset detection as compared to several previously reported approaches, particularly with respect to extreme misestimations. These findings are robust against at least some language- and speaker-specific influences in standard laboratory settings, as demonstrated by our tests of performance on two distinct datasets and languages. Of course, additional work remains to establish the breadth to which the present features generalize. This is a critical and fundamentally empirical question—which is also typically not even asked in studies of automatic speech onset detection.

To facilitate answering this question, we have provided an easy-to-use Web platform, as well as the full source code for Chronset, for use by other researchers. These tools should also enable the rapid reoptimization of Chronset’s parameters if other data sources are discovered in which performance is suboptimal, as may be the case with some specialized populations (e.g., onset detection in children or patient populations).

As we highlighted by our “mixture-of-experts” algorithm, the availability of our source code and an automated platform for onset estimation may also be useful for combining the unique strengths and overcoming the weaknesses of multiple estimation algorithms, to improve performance above the levels of Chronset (or any other algorithm’s) performance in isolation. Such comparisons may also be especially fruitful in identifying the areas of a particular algorithm that may benefit from targeted improvement. Indeed, the detailed comparisons that we conducted between Chronset and of the current standard models in the field have helped point us toward improving Chronset’s onset sensitivity within the < 50 ms range. Future work will evaluate whether improvements in this window can be achieved through a more extensive and computationally intense optimization of the parameters governing the temporal smoothing that helps Chronset achieve robust overall patterns of performance, and through the incorporation of additional features into the current feature set.

### Using Chronset

Chronset is available for public use under the GNU General Public License through the Chronset website (www.bcbl.eu/databases/chronset), either through a Web interface or by downloading and running the source code. To estimate speech onset latencies automatically via the Chronset website, speech recordings are required to be uploaded to the website in.wav format. Once the files are uploaded, they will be processed using Chronset (average processing time per.wav file of ~1–15 s, depending on the server load). The resulting onset latencies will be sent via email message once Chronset has terminated processing each file. Using the source version of Chronset, it is also possible to use parallel computing to process multiple files simultaneously.

## Electronic supplementary material

Below is the link to the electronic supplementary material.

ESM 1 (PDF 147 kb)
