# Supplementary material for: Chronset: An automated tool for detecting speech onset
Source: Behav Res Methods. 2016 Dec 6;49(5):1864–81. doi: 10.3758/s13428-016-0830-1 (PMC5628189; doi:10.3758/s13428-016-0830-1)
Supplement: Supplementary file 1 — (PDF 147 kb) [file 13428_2016_830_MOESM1_ESM.pdf]

Chronset: Supplementary Information

Additional details regarding human onset detection

For each data set, speech onset latencies were measured by a group of human raters. In the case of the data set 2, ratings from one human were already available; these were supplemented by data from two additional raters who manually identified onsets in the SayWhen data after it had been split into separate data files for each trial. The two additional raters performed this task using the Audacity software, whereas the original rater used the SayWhen software. For data set 1, data from two raters were available.

Detailed comparison of onset latencies determined by human raters

A core assumption in the literature is that the most precise way to measure voice onset latency is by having a human rater examine the waveform and/or spectrogram. This has led to the adoption of human ratings as the gold standard against which automatic detection algorithms are compared. Given how well Chronset is able to account for the latency data, as well as the lack of a perfect one-to-one match between different human raters, the degree to which the human ratings should be treated as a gold standard when automated detection algorithms approach asymptotic performance becomes an increasingly important question.

To determine the degree to which the data from human raters should be treated as a gold standard, we conducted a detailed comparison between the performance of two human raters on our version of the SayWhen dataset (which is identical to the original dataset, except that individual trials were spliced out of the original continuous recording). One of these raters was a professional research assistant with several years of training on tasks that included speech onset detection based on visual inspection of both the waveform and the spectrogram of a recording. The second rater was a new research assistant with no experience with the task. A comparison between these two raters therefore provides insight not only into the level of agreement between raters, but how rating agreement could vary as a function of experience with the task.

Prior to beginning the experiment, the new research assistant was instructed in how to identify onset latencies based on the waveform and spectrogram by the trained research assistant and the second author. Both research assistants then coded the data from the first participant in the dataset. The resulting ratings were inspected by both research assistants and the second author as a group to identify the source of any discrepant ratings and to improve agreement. These data were then discarded and both research assistants coded the data from each participant in order.

Figure S1 plots the latencies identified by each rater, as well as the regression slope that best fit these data. Overall, there was a high level of agreement, although there were some discrepancies that appeared to be more common for some participants than others. To quantify these discrepancies and gain finer-grained insight into the agreement levels between the raters, we extracted the slope, intercept, and degree of fit (r_2) for each participant, as determined by each rater, and then plotted how these values changed as a function of experience completing the rating task (here reflected by participant number, with coding starting from the smallest number). Figure S2, S3, and S4, plot the agreement levels for each of these parameters. In all cases, the regression parameters were relatively stable, showing little change as a function of experience. This suggests that an initial training session by experienced raters is sufficient for new research assistants to produce high-quality estimates of latency onsets. However, there is one caveat --- whereas the near-one value of the slope indicates and of the percent of shared variance that rater agreement was similar for both fast and slow trials, the intercept was offset systematically by 17 ms. This suggests that, at least for our data, the raters were biased in the amount of evidence that was needed before they would indicate that a vocal response had been made. This finding has important ramifications for how automatic algorithms should be evaluated relative to human data. Given that different humans show high levels of overall agreement but, at least in our data, may also be systematically offset, comparing the gold standard to an algorithm is more appropriate in terms of relative agreement rather than in terms of absolute values, because the gold standard does not actually provide precise estimates of the onset itself. For this reason, we evaluated Chronset's performance relative to the human raters by focusing on the overall level of agreement, as expressed by the standard deviation of the residual variance in a regression between human ratings and the different algorithms.

For Review Only

Rationale for the threshold method for identifying onsets using multiple acoustic features

In designing Chronset, we considered several different possible methods for how the thresholds could be used to identify onsets before adopting the method reported in the main text. One simple approach that is particularly worthy of discussion was why we did not simply sum the values of each of the different features and establish a single threshold value that, once exceeded, would denote the onset of speech. This was because, in practice, such an approach leaves to be desired because the difference between speech and noise often occurs in a different range of values for different features. Additionally, the change between speech and nonspeech may be nonlinear, which would make the use of a simple (linear) sum of feature values inappropriate. To address this issue, we established different threshold values for each feature which delineated between speech and nonspeech.

Optimization of thresholds for onset detection

To determine when speech occurs Chronset requires that a set of thresholds are crossed simultaneously for at least four different acoustic features. To ensure the robustness of the thresholds estimated in the present study, we estimated the thresholds for each individual feature by employing a gradient descent approach which builds on neural network optimization techniques and other related methods (e.g., Hinton & Sejnowski, 1986; Armstrong, Watson, & Plaut, 2012; Jacobs, 1988; Metropolis-Hastings search---Hastings 1970). Prior to optimization, the recorded speech waveforms were divided into a training set (80%) and a testing set (20%). Optimization was performed on the training set, while the testing set was used to assess the robustness of the features (feature thresholds were set to an initial set of values: 0.1 for all features except WE and FM, which were set to 0.9, effectively requiring little evidence of a speech-like signal to cross a feature threshold; See Figure 5a). The overall fit provided by the estimated thresholds was calculated by comparing human latencies against the latencies estimated by Chronset. A poor fit of the estimated thresholds was associated with a higher standard deviation (SD) of differences between human and automatic latencies, whereas a better fit is associated with a lower SD. With this measure, it is possible to optimize the thresholds by selecting those thresholds that lower the overall SD.

To optimize the individual feature thresholds, we first randomly selected one of the six features and then modified this feature by adding and subtracting a fixed value *vf* (initial value of 0.05) from the current threshold specific to each feature. We then compared the SD associated with each of those sets to that obtained on the basis of the initial values of the thresholds. If one of the modified threshold sets yielded a lower SD than the thresholds prior to modification, the default set of thresholds was updated with this new set; otherwise, the default set of thresholds was retained. To accelerate the gradient descent process, if the default set was changed the value of *vf* was also increased by multiplying the current value by a rate increment of 1.1. This ensured that the optimization was able to move away from poor threshold values rapidly. In contrast, if the default set was not changed this could be due to the target threshold being close to an optimal value, such that a large increase or decrease made the overall fit of the data worse. For this reason, if the default threshold did not change, vf was decreased by multiplying the current value by a rate decrement of 0.5 (see Figure 5c for the values of vf as a function of number of iterations). Note that rates decreased faster than they increased because smaller threshold changes in the correct direction will always improve the set, albeit slowly, whereas larger threshold changes do not improve the set.

The optimization algorithm attempted 1000 iterations of updating the threshold values before stopping, or stopped after 50 successive iterations had failed to alter the thresholds. Inspection of SD as a function of total number of iterations indicated that a low SD plateau had been reached at this point and the values of vf for each of the thresholds were very small; both indicators that an optimized set of thresholds had been reached. The overall fit of the thresholds was then measured by comparing the human ratings against automatic latencies for the test data. To maximize the likelihood that the best possible thresholds were identified, we repeated this optimization process for 200 different partitions of training and test trials. The final set of thresholds were those associated with the smallest SD on the testing data (Figure 5d).

For Review Only

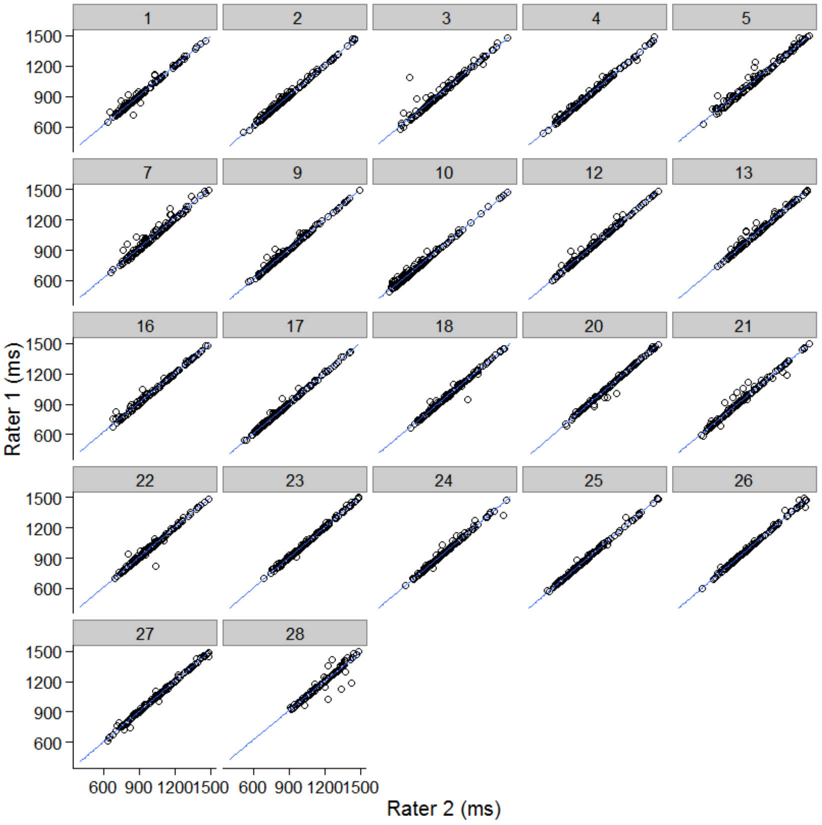

S1. Scatterplots of the latencies identified by rater 1 (x-axis) and rater 2 (y-axis) for each participant (identified by their participant code) in the data set with more than **150** observations.

view Only

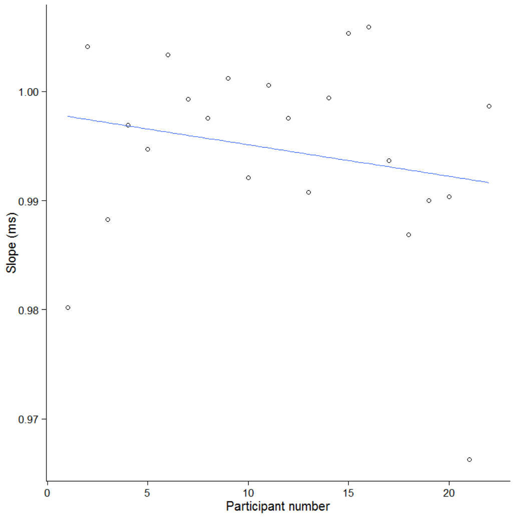

Figure S2. Slope of the regression between the raters as a function of experience

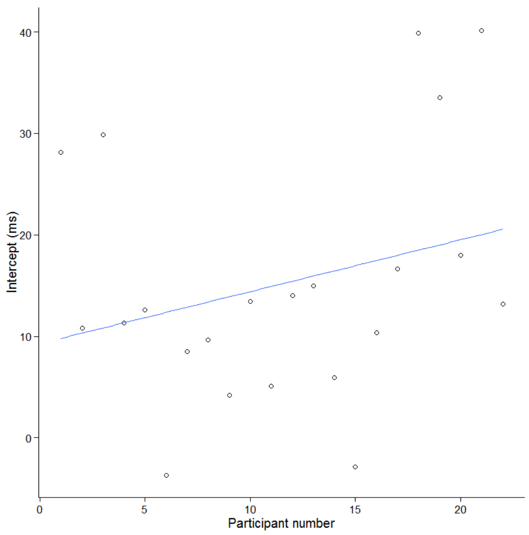

Figure S3. Intercept of the regression between the raters as a function of experience.

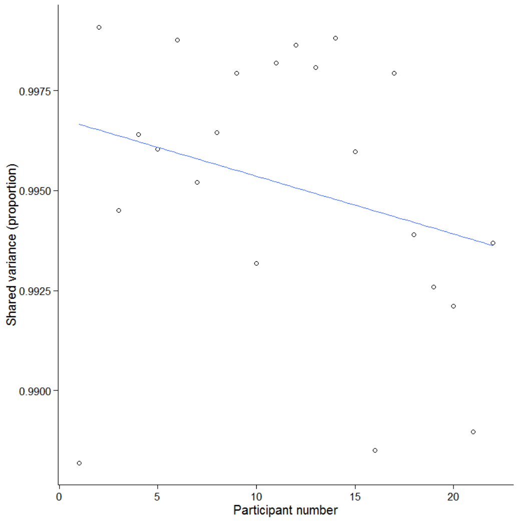

Figure S4. Percent of variance shared (r_2) across the ratings of both raters as a function of experience.

For Review Only

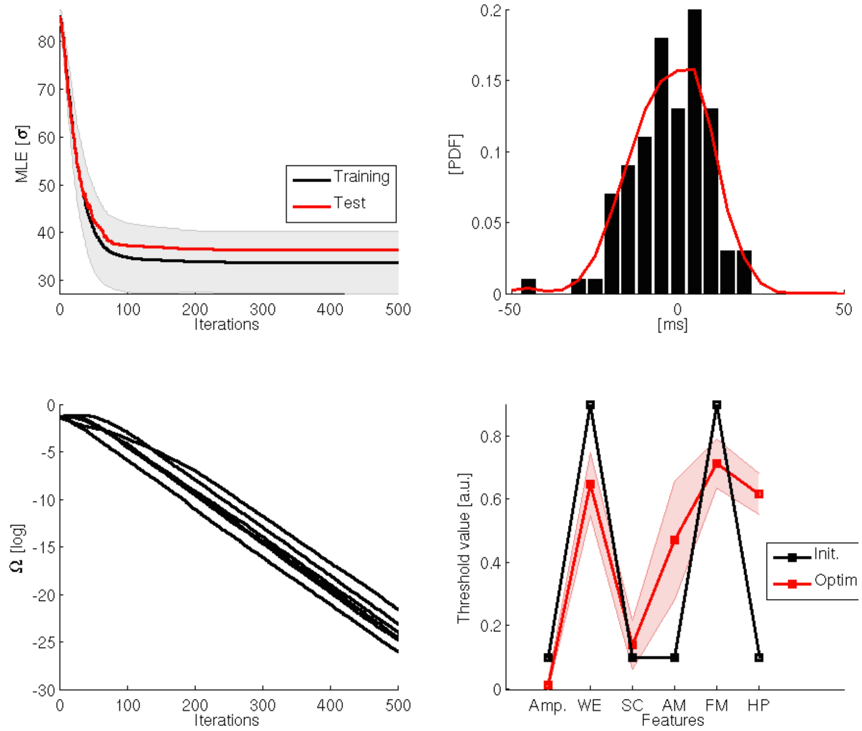

Figure S5. [upper left] Plot of the standard deviation from the MLE for the training data (black) and test data (red) as a function of number of iterations in the optimization. [upper right]. MLE distribution for the test data set. [lower left]. Size of the change (increment & decrement) for each feature threshold as a function of number of iterations in the optimization. [lower right]. Plot of the initial feature thresholds (black) and optimized feature thresholds (red).

Review Only
